# MAKER2: an annotation pipeline and genome-database management tool for second-generation genome projects

**DOI:** 10.1186/1471-2105-12-491

**Published:** 2011-12-22

**Authors:** Carson Holt, Mark Yandell

**Affiliations:** 1 Eccles Institute of Human Genetics, University of Utah, Salt Lake City, Utah 84112, USA; 2 Ontario Institute for Cancer Research, MaRS Centre, South Tower 101 College Street, Suite 800 Toronto, Ontario, Canada M5G 0A3

## Abstract

**Background:**

Second-generation sequencing technologies are precipitating major shifts with regards to what kinds of genomes are being sequenced and how they are annotated. While the first generation of genome projects focused on well-studied model organisms, many of today's projects involve exotic organisms whose genomes are largely *terra incognita*. This complicates their annotation, because unlike first-generation projects, there are no pre-existing 'gold-standard' gene-models with which to train gene-finders. Improvements in genome assembly and the wide availability of mRNA-seq data are also creating opportunities to update and re-annotate previously published genome annotations. Today's genome projects are thus in need of new genome annotation tools that can meet the challenges and opportunities presented by second-generation sequencing technologies.

**Results:**

We present MAKER2, a genome annotation and data management tool designed for second-generation genome projects. MAKER2 is a multi-threaded, parallelized application that can process second-generation datasets of virtually any size. We show that MAKER2 can produce accurate annotations for novel genomes where training-data are limited, of low quality or even non-existent. MAKER2 also provides an easy means to use mRNA-seq data to improve annotation quality; and it can use these data to update legacy annotations, significantly improving their quality. We also show that MAKER2 can evaluate the quality of genome annotations, and identify and prioritize problematic annotations for manual review.

**Conclusions:**

MAKER2 is the first annotation engine specifically designed for second-generation genome projects. MAKER2 scales to datasets of any size, requires little in the way of training data, and can use mRNA-seq data to improve annotation quality. It can also update and manage legacy genome annotation datasets.

## Background

Second-generation sequencing technologies are creating new opportunities as well as new challenges for genomics research. While first-generation genome projects focused primarily on established model organisms such as *Drosophila melanogaster*[1], *Caenorhabditis elegans*[2], and *Mus musculus*[3], falling sequencing costs are allowing second-generation genome projects to focus on more exotic and phylogenetically isolated organisms. The large volumes of data produced by second-generation sequencing technologies also create difficulties for data management not encountered by first-generation projects. Together, these factors can spell disaster for second-generation genome projects.

The first-generation of genome projects benefited greatly from large bodies of pre-existing knowledge regarding their organisms' genomes. For *D. melanogaster*, *C. elegans*, and *Homo sapiens*[4,5], for example, hundreds of published gene models already existed before these genomes were sequenced. These pre-existing published gene models were critical for annotation and analysis, because they allowed researchers to train and optimize gene prediction and annotation tools for each genome. They also provided a standard by which to judge the accuracy of annotations. Second-generation projects rarely have access to such information. This severely limits their ability to train *ab initio *gene-finders, the result being (as we show below) low-quality gene predictions. The lack of pre-existing gene models also leaves many second-generation projects with no objective standards with which to gauge annotation accuracy. Quality control is thus a significant issue for these projects; data management is another.

New techniques such as high-throughput transcriptome sequencing (mRNA-seq) have great potential to improve annotation quality, but they produce enormous amounts of data; likewise, the existence of legacy annotations for a large number of both first and second-generation genomes is also creating data management challenges. Thus, it is essential that the output of an annotation pipeline be easily converted into a genome database.

MAKER2 builds upon MAKER[6], an easy-to-use genome annotation pipeline that has seen wide adoption[7-19]. MAKER2 improves upon the *de novo *annotation capabilities of the original MAKER and integrates support for multiple *ab initio *prediction tools. Major additions to MAKER2 include integration of the Annotation Edit Distance (AED)[20] metric for improved quality control and downstream database management, support for mRNA-seq to allow researchers to leverage second generation sequencing technologies, and gene model pass-through capability; thus creating a first of it's kind tool for updating and reannotating existing model organism databases. The pipeline also supports distributed parallelization on computer clusters via MPI which means MAKER2 can scale to datasets of virtually any size. MAKER2 can run on UNIX-like operating systems such as Linux and Darwin in Mac OS X. MAKER2 thus provides straightforward solutions to the problems facing today's second-generation genome projects. Here, we demonstrate MAKER2's ability to overcome handicaps resulting from a lack of pre-existing gene models with which to train gene-predictors for use on novel genomes; its ability to use mRNA-seq data to improve annotation quality; and its ability to update legacy annotations and in doing so significantly improve their quality.

MAKER2 is not only an improved annotation engine; it is also a new kind of annotation management tool. Throughout these analyses, we measure MAKER2's performance using the integrated annotation quality-control measure AED, developed by the Sequence Ontology project[21]. We show that MAKER2 can both evaluate the global quality of genome annotations, and identify and prioritize problematic annotations for manual review; these are functionalities offered by no other annotation tool.

## Implementation

### *De novo *annotation of first-generation genomes

*D. melanogaster *chromosome 3R and GFF3 annotations for release r5.32 were downloaded from FlyBase. *C. elegans *chromosome V and GFF3 annotations for release WS221 were downloaded from WormBase. *Arabidopsis thaliana*[22] chromosome 4 and GFF3 annotations were downloaded from TAIR. Each set of reference gene annotations were passed to MAKER2's model_gff option with all prediction and evidence alignment options turned off. This has the effect of repackaging the reference gene models into more standardized GFF3 files compatible with downstream analysis scripts.

*Ab initio *gene predictions were produced by the programs SNAP[23] version 2010-07-28, Augustus[24] 2.5.5, and GeneMark-ES[25] 2.3a, using the *D. melanogaster*, *C. elegans*, and *A. thaliana *parameter files pre-packaged with each algorithm (GeneMark parameter files are packaged with the GeneMark.hmm download). To produce all predictions in standardized GFF3 format, these algorithms were run through MAKER2 with all evidence alignments options turned off and the keep_preds flag set to 1. This has the effect of only producing raw *ab initio *gene predictions in standardized GFF3 format.

Evidence-based gene annotations in MAKER2 were produced using default settings. The species parameter files were the same as those used for the *ab initio *gene-predictors. EST and protein homology datasets were provided for each organism. For *D. melanogaster*, the EST dataset consisted of all *D. melanogaster *ESTs available from dbEST[26], and the protein homology input consisted of all *Anopheles gambiae*[27] proteins from NCBI together with all of the UniProt/Swiss-Prot[28,29] database proteins (minus Drosophila proteins). For *C. elegans*, the EST dataset consisted of all *C. elegans *release WS221 ESTs available from WormBase, and protein homology input consisted of all *Caenorhabditis briggsae*[30] WS221 proteins from WormBase together with all of the UniProt/Swiss-Prot database proteins (minus Caenorhabditis proteins). The EST dataset for *A. thaliana *consisted of all *A. thaliana *ESTs from dbEST, and the protein homology dataset consisted of all *Oryza sativa*[31] release 6.1 proteins from PlantGDB and all of the UniProt/Swiss-Prot database proteins (minus Arabidopsis proteins). For *A. thaliana*, MAKER2 was also provided with the Arabidopsis transposable element FASTA file available from TAIR (assists in repeat masking).

The reference gene models, *ab initio *gene predictions, and evidence-based gene annotations were converted to GTF format using the maker2eval script packaged with MAKER2. Values for sensitivity and specificity were then produced using Eval[32].

### *De novo *annotation using unmatched species parameters

To simulate non-optimal training of the *ab initio *gene-finders, *ab initio *predictions and MAKER2 annotations were produced for *D. melanogaster*, *C. elegans*, and *A. thaliana *using unmatched species parameter files. This was done by running SNAP, Augustus, GeneMark, and MAKER2 on *C. elegans *and *D. melanogaster *using the *A. thaliana *parameter files. These programs were then run on *A. thaliana *using the *C. elegans *parameter files. All other steps and procedures were identical to the previous analysis.

### *De novo *annotation of second-generation genomes

*Schmidtea mediterranea*[17] assembly 3.1 and *Linepithema humile*[9] assembly 4.0 were used to evaluate the performance of the *ab initio *gene-predictor SNAP and the annotation pipeline MAKER2 on second-generation genome projects. To produce SNAP required parameter files for each species, we first ran CEGMA[33], which produces gene models that can be used for training SNAP from a core set of universal genes that should be found in all eukaryotes. CEGMA gene models were converted to SNAP's required ZFF format using the cegma2zff script that comes bundled with MAKER2. SNAP was then trained in accordance with its documentation. To produce all predictions in standardized GFF3 format, SNAP was run via MAKER2 with all evidence alignments options turned off and the keep_preds flag set to 1. This has the effect of only producing raw *ab initio *gene predictions in standardized GFF3 format.

MAKER2 was run on *S. mediterranea *using an EST dataset consisting of all ESTs available for *S. mediterranea *found in dbEST together with the SmedGD EST dataset. The protein homology dataset consisted of all proteins in the UniProt/Swiss-Prot protein database, all *Schistosoma mansoni*[34] v4.0 proteins from Sanger, and all GenBank[35] proteins for *Nematostella vectensis*, *H. sapiens*, *C. elegans*, and *S. mediterranea*. The SmedGD repeat library was also used. Short read mRNA-seq transcriptome datasets for *S. mediterranea *were downloaded from the NCBI Sequence Read Archive (SRP006000). TopHat[36] v1.2.0 and Cufflinks[37] v0.9.3 were used to align and process these short reads. The script tophat2gff3 and cufflinks2gff3 were then used to process the results into GFF3 format. The resulting GFF3 files were provided to the est_gff option in MAKER2.

MAKER2 was run on *L. humile *using the published genome project EST dataset together with all Apocrita and Formicidae ESTs available from dbEST. The protein homology dataset consisted of all of the UniProt/Swiss-Prot protein database, *D. melanogaster *r5.32 proteins, *Nasonia vitripennis*[38] OGS 1.2 proteins, *Apis mellifera*[39] OGS 2 proteins, and all Formicidae proteins from GenBank. A combined repeat FASTA file from the published *L. humile *and *Pogonomyrmex barbatus*[8] genomes was also provided.

### Pfam domain analysis

InterProScan[40] was used to identify Pfam[41] domains for all gene prediction/annotation datasets. Domains were filtered to remove reverse transcriptase, integrase, and virus related protein domains. Any domain listed as unknown, uncharacterized, or NULL was ignored.

To explore the upper bound of expected Pfam domain content in a newly annotated genome, we used InterProScan to identify Pfam protein domains in *H. sapiens *release 37.2, *M. musculus *release 37.1, *D. melanogaster *r5.32, *C. elegans *WS221, and *Saccharomyces cerevisiae*[42] (NCBI release). Domains were filtered as before (i.e. remove reverse transcriptase, integrase, and virus related domains). The average domain enrichment for these reference genomes was then calculated for comparison.

### Calculating Annotation Edit Distance

Sensitivity, specificity, and accuracy are commonly used metrics for evaluating the performance of gene prediction algorithms by comparing the resulting gene prediction to a well-supported reference annotation[43]. Sensitivity is defined as the fraction of a reference overlapping a prediction; specificity is defined as the fraction of a prediction overlapping a reference; and accuracy is commonly defined as the average of sensitivity and specificity (although several alternate formulations exist). Both sensitivity and specificity can be calculated for any feature in the genome at different levels of stringency (i.e. base pair level, exon level, etc.).

Given a gene prediction *i *and a reference *j*, the base pair level sensitivity can be calculated using the formula *SN = |i∩j|/|j|*; where *|i∩j| *represents the number overlapping nucleotides between *i *and *j*, and *|j| *represents the total number of nucleotides in the reference *j*. Alternatively, specificity is calculated using the formula *SP = |i∩j|/|i|*, and accuracy is the average of the two.

When calculating AED, we adapt the calculation of sensitivity and specificity to account for the fact we do not have a reference gene model for comparison; instead, we cluster experimental evidence aligned against the genome to approximate the reference. So for *SN = |i∩j|/|j|*, the value *|i∩j| *represents the number of nucleotides in a gene prediction overlapped by experimental evidence, and *|j| *represents the total base pair count for experimental evidence in that cluster. Because we are not comparing to a high quality reference, it is more correct to refer to the average of sensitivity and specificity as the *congruency *rather than accuracy; where *C = (SN+SP)/2*. The *incongruency*, or distance between *i *and *j*, then becomes *D = 1-C*, with a value of 0 indicating complete agreement of an annotation to the evidence, and values at or near 1 indicating disagreement or no evidence support.

### AED evaluation for the human and mouse genomes

*H. sapiens *annotations for releases 33 and 37.2 as well as *M. musculus *annotations for releases 30 and 37.1 were downloaded from NCBI in GenBank file format. They were converted to GFF3 format using the genbank2gff3 script available in the BioPerl[44] 1.6 distribution. The resulting GFF3 files were passed to MAKER2's model_gff option with all prediction and evidence alignment options turned off. This has the effect of repackaging the gene models into more standardized GFF3 files compatible with downstream analysis scripts.

The standardized GFF3 files were then provided to MAKER2's model_gff option once again together with protein and EST datasets to produce downstream quality control metrics for each gene model. The human reference gene annotations were processed using all human ESTs from dbEST and a protein dataset consisting of all mouse proteins together with all of UniProt/Swiss-Prot (minus human proteins), and the genome was masked using the mammal subset of repeats from RepBase[45]. The mouse reference gene annotations were processed using all mouse ESTs from dbEST and a protein dataset consisting of all human proteins together with all of UniProt/Swiss-Prot (minus mouse proteins), and the genome was masked using the mammal subset of repeats from RepBase.

The presence/absence of human release 33 genes in release 37.2 and mouse release 30 genes in release 37.1 was determined using BLASTP[46] and reciprocal best hits analysis (where genes from each dataset are each others best hit). A threshold e-value of 1 × 10^-6 ^was required for all hits. Pfam domains were also mapped to all genes using the previously described methodology.

### Re-annotation of the maize genome

To demonstrate MAKER2's ability to re-annotate existing genomes with respect to legacy annotations, we re-annotated a 22 megabase region of the *Zea mays *(maize) inbred line B73 chromosome 4[47], available from http://maizesequence.org. We then used the subset of reference annotations that are also included in the Maize Classical Gene List[48] as a 'gold standard' set to evaluate MAKER2's performance.

We first produced a standardized GFF3 file for the maize reference annotations by using the map2assembly script bundled with MAKER2 to map maize reference transcripts onto the genome. We then provided the resulting GFF3 file to MAKER2 via the model_gff option and provided an EST dataset consisting of all ESTs/cDNAs for maize available from the Maize Full Length cDNA Project[49] and dbEST. The protein homology dataset we used consisted of the *A. thaliana *proteome and all of the UniProt/Swiss-Prot database (minus any maize proteins). Maize specific repeats were acquired from the Maize Transposable Element Database[50]. The resulting MAKER2 output was a GFF3 file containing AED quality control values for all reference transcripts. The AED distribution of the reference was then graphed together with the AED distribution for the 'gold standard' genes identified as overlapping the Maize Classical Gene List.

Next we produced *de novo *annotation and a re-annotation dataset using MAKER2. The *de novo *annotation dataset was produced using the maize prediction parameter file that comes bundled with SNAP. We also provided MAKER2 with the same EST, protein, and repeat datasets used in the previous analysis. To produce the re-annotation dataset, we again used the same EST, protein, repeat, and SNAP files; however, we also passed MAKER2 all legacy annotations by indicating the location of the reference GFF3 file in the model_gff option. We then graphed the AED distributions as was done previously for the reference dataset.

### Evidence alignment and analysis of published ant genomes

To demonstrate how MAKER2 can be used to add experimental evidence and quality control statistics to existing genome databases (which can fuel downstream analyses or be used to improve annotations), we used MAKER2 to add cross-species homology data to six published ant genomes. We downloaded annotations for *Atta cephalotes*[7] OGS 1.2, *L. humile *OGS 1.2, *P. barbatus *OGS 1.2, *Camponotus floridanus*[51] v3.3, *Harpegnathos saltator*[51] v3.3, and *Solenopsis invicta*[18] v2.2.0 from the Hymenoptera Genome Database[52]. Most species had GFF3 format annotations that were passed to MAKER2's model_gff option, with all prediction and evidence alignment options turned off. This has the effect of repackaging the gene models into more standardized GFF3 files compatible with downstream analysis scripts. For *S. invicta*, however, we used the map2assembly script bundled with MAKER2 to map transcripts onto the genome assembly (thus producing a standardized GFF3 formatted annotation file).

We next ran MAKER2 on each of the six ant species. Standardized GFF3 files were passed to MAKER2's model_gff option. We used an EST dataset consisting of all Apocrita and Formicidae ESTs available from dbEST (filtered to not include ESTs for the species being analyzed). We used a protein homology dataset consisting of all of the UniProt/Swiss-Prot protein database, *D. melanogaster *r5.32, *N. vitripennis *OGS 1.2, *A. mellifera *OGS 2, and all of the published ant proteomes (always excluding the species being processed at the time). A combined ant repeat FASTA file from the published *L. humile *and *P. barbatus *genomes was also provided.

Orthology of the six ant species was explored using BLASTP and reciprocal best hits analysis. A threshold e-value of 1 × 10^-6 ^was required for all hits. We also used InterProScan to identify Pfam domains for all proteins using the previously described methodology.

### Evaluation of high through-put parallelization

The parallelization performance of MAKER2 was evaluated on a server with four, twelve-core AMD Opteron 6174 Processors (48 total CPU cores) running Red Hat Enterprise Linux Server release 5.5. MAKER2 was configured with default settings and the NGASP[53] protein, EST, and genomic sequence datasets available from WormBase. The NGASP genomic sequence is a selected 10 megabase sampling of the *C. elegans *genome (release WS160). We ran MAKER2 (the parallel executable is mpi_maker) using 1, 4, 8, 16, and 32 CPU cores under MPICH2 1.3.1. The Linux time command was used to evaluate process run time.

## Results and Discussion

### Genome annotation in model organism genomes

The performance of *de novo *annotation tools such as HMM based *ab initio *gene-predictors and evidence based annotation pipelines have previously been explored in competitions such as EGASP[54] and NGASP, which looked at gene prediction and annotation accuracy in the human and *C. elegans *genomes, respectively. From these competitions, the metrics sensitivity, specificity, and accuracy have emerged as the standard methods for evaluating the quality of gene predictions[55]. These measurements require a set of reference gene models that are assumed to be correct, as gene predictions are compared to the reference models to generate sensitivity, specificity and accuracy values (see Implementation section).

For reference purposes, we first compared the performance of MAKER2 to the *ab initio *gene prediction programs SNAP, GeneMark, and Augustus on three different first-generation genomes. We used the organism specific parameter files that come bundled with each of these algorithms to produce *ab initio *gene predictions for *D. melanogaster *chromosome 3R, *C. elegans *chromosome 5, and *A. thaliana *chromosome 4. For comparison, we then produced evidence-based genome annotations by running the same three algorithms (SNAP, GeneMark, and Augustus) inside of the MAKER2 genome annotation pipeline. Sensitivity, specificity, and accuracy values were then calculated against the respective reference genome using the program Eval (Table 1 and Additional file 1 Table S1).

**Table 1:** Gene model accuracy for gene prediction/annotation programs

Reference Organism	Performance Category	*Ab Initio *Predictions			MAKER Annotations		
		
		Augustus	GeneMark	SNAP	Augustus	GeneMark	SNAP
*A. thaliana*	Nucleotide Accuracy	77.04%	74.68%	69.78%	80.53%	79.39%	80.27%
	Exon Accuracy	67.03%	61.31%	56.40%	67.81%	69.60%	68.78%
*D. melanogaster*	Nucleotide Accuracy	76.08%	66.54%	69.29%	76.42%	73.66%	74.33%
	Exon Accuracy	61.37%	47.31%	47.01%	58.56%	58.03%	58.49%
*C. elegans*	Nucleotide Accuracy	88.29%	88.09%	85.10%	87.14%	86.29%	88.48%
	Exon Accuracy	74.62%	68.88%	61.38%	68.60%	65.03%	66.19%

Comparison of gene model accuracies in first-generation genomes for the *ab initio *gene predictors Augustus, GeneMark, and SNAP in comparison to gene model accuracies produced by the same predictors when ran as part of the MAKER2 gene annotation pipeline.

As seen in Table 1, the base pair and exon level accuracy values for *ab initio *predictions produced by SNAP, Augustus, and GeneMark are very similar, generally within a few percentage points of each other. In *C. elegans*, for example, the difference between low and high base pair level accuracies is only 3.19% (85.10% for SNAP vs. 88.29% for Augustus). The corresponding MAKER2 annotations have similar accuracies relative to the *ab initio *gene predictions, and more often than not, they are slightly improved over the *ab initio *gene predictions, but the improvements are small. In *C. elegans*, for example, base pair level accuracies in MAKER2 range from 86.29% to 88.48% which is comparable to the 85.10% to 88.29% range for the *ab initio *gene predictions. This is not the first time that this trend has been observed[53] -- given large enough training sets, *ab initio *gene prediction programs can match or even outperform annotation pipelines. Augustus, for example, achieved an exon-level accuracy in *C. elegans *of 74.62%, compared to MAKER2's 68.60% (Table 1).

The relative similarity of accuracy measurements for *ab initio *prediction methods vs. MAKER2 suggests that MAKER2 is performing on par with these *ab initio *tools (but not greatly improving accuracy). However, as we show below, such comparisons can be quite misleading from a second-generation genome perspective. The key to understanding why is grasping that Table 1 reports the performance of the *ab initio *predictors after they have been trained using each genome's existing annotations -- datasets containing tens of thousands of often hand-curated gene models. Data such as those shown in Table 1 thus represent the upper bounds for performance of the *ab initio *prediction algorithms. As we demonstrate below, when training sets decrease in quality and/or size, the accuracy of *ab initio *tools drops dramatically; in contrast MAKER2's accuracy, however, remains high. This feature of MAKER2 makes it especially useful for second-generation genome projects as these projects generally lack large enough training datasets for *ab initio *predictors to achieve accuracies comparable to those shown in Table 1.

### Genome annotation using unmatched species parameters

To better understand how these algorithms perform using poor quality training data, we repeated our analysis shown in Table 1 using the same portions of *D. melanogaster *chromosome 3R, *C. elegans *chromosome 5, and *A. thaliana *chromosome 4; but this time we intentionally ran the gene-predictors using the wrong species file for each organism. *D. melanogaster *and *C. elegans *were analyzed using the parameter file for *A. thaliana*, and *A. thaliana *was analyzed using the parameter file from *C. elegans*. Each *ab initio *gene prediction program was then run inside of the MAKER2 annotation pipeline using the same incorrect parameter files for comparison.

As expected, the accuracy of the *ab initio *prediction algorithms is reduced substantially (Table 2 and Additional file 1 Table S2). The reduction in accuracy is most notable at the exon level where all accuracies were approximately half of what was seen in the previous analysis shown in Table 1. However, when each *ab initio *prediction program was run inside of MAKER2, accuracies dramatically improved for every organism at both the base pair and exon levels. The degree of improvement was most notable for SNAP, where exon level accuracies for *A. thaliana *increased from 18.58% to 60.11%. In fact, SNAP's performance inside of MAKER2 using the incorrect parameter files often matched or even exceeded the levels of performance delivered by all three *ab initio *gene-predictors when run using the correct parameter files. For example for *D. melanogaster*, when using the incorrect SNAP parameter file, MAKER2 produces exon level accuracies of 53.69%; whereas when using the correct parameter files outside of MAKER2, the programs GeneMark, SNAP, and Augustus produce exon level accuracies of 47.31%, 47.01%, and 61.37%, respectively. These data show that MAKER2 can substantially improve the performance of *ab initio *gene-predictors in situations where training data may be of poor quality.

**Table 2:** Gene model accuracy using unmatched species parameters

Reference Organism	Performance Category	*Ab Initio *Predictions			MAKER Annotations		
		
		Augustus	GeneMark	SNAP	Augustus	GeneMark	SNAP
*A. thaliana*	Nucleotide Accuracy	57.85%	48.62%	43.84%	68.56%	57.96%	73.77%
	Exon Accuracy	30.71%	16.51%	18.58%	53.31%	28.87%	60.11%
*D. melanogaster*	Nucleotide Accuracy	67.47%	66.51%	48.92%	73.78%	72.83%	74.44%
	Exon Accuracy	30.62%	26.25%	19.94%	43.10%	39.74%	53.69%
*C. elegans*	Nucleotide Accuracy	66.18%	67.26%	68.24%	74.32%	71.92%	85.02%
	Exon Accuracy	28.33%	30.01%	35.44%	38.52%	39.42%	63.14%

The effect of limited/insufficient training data on *ab initio *gene prediction is simulated by providing the algorithms Augustus, GeneMark, and SNAP with incorrect species parameters files (the *A. thaliana *species parameters were used to produce gene models for *C. elegans *and *D. melanogaster*, and the *C. elegans *parameters were used to produce gene models in *A. thaliana*). In comparison, the same predictors, when ran as part of the MAKER2 gene annotation pipeline, perform substantially better, even with the same incorrect species parameter files.

### Gene prediction/annotation in second-generation genomes

When analyzing the performance of gene-predictors in sequenced second-generation genomes, the same metrics of sensitivity, specificity, and accuracy used for first-generation genomes cannot be applied (Table 1 and Additional file 1 Table S1). This is because second-generation genomes lack the high-quality reference gene models required to calculate these values (accuracy measures the overlap between a prediction and the supposed correct reference).

In the experiments below, we use Pfam domain content (mapped using InterProScan) as a proxy metric for annotation quality. Although expansion and contraction of gene families can be an important mode of organism evolution, previous work has shown that the high level of domain content of eukaryotic proteomes is relatively invariant[56]; this fact can be clearly seen in Additional file 1 Table S3, which documents the high-level Pfam domain frequencies for six different well annotated eukaryotic model organisms (*H. sapiens*, *M. musculus*, *D. melanogaster*, *C. elegans*, *A. thaliana*, and *S. cerevisiae*). Thus at the grossest level of resolution, the percentage of annotations containing one or more Pfam domains provides an indication of annotation accuracy.

**Figure 1:**
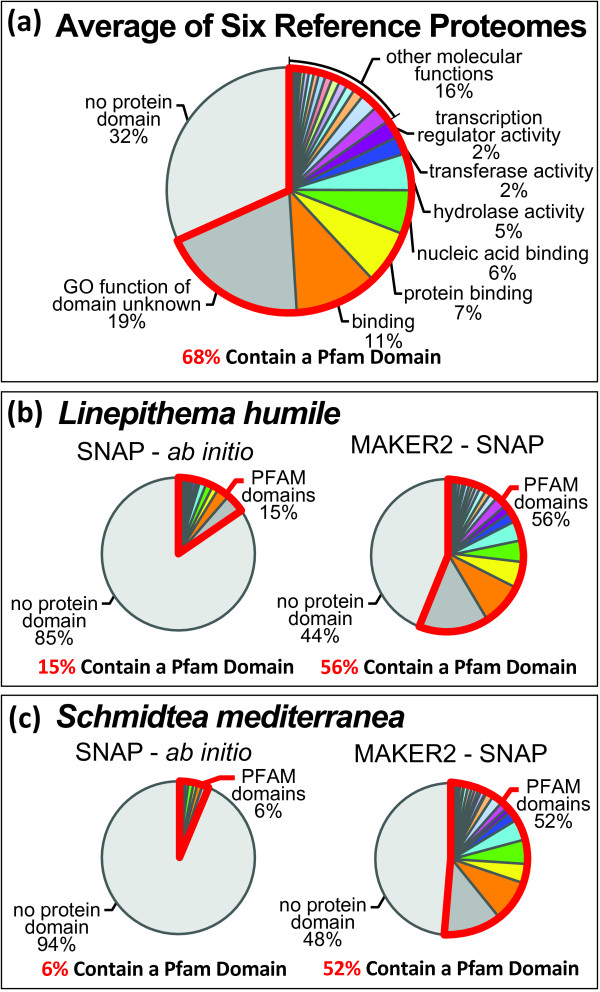
**MAKER2 vs. *ab initio *predictors on second-generation genomes**. We compared the performance of the *ab initio *predictor SNAP to the annotation pipeline MAKER2 on two second-generation genomes: *L. humile *(Argentine ant) and *S. mediterranea *(flatworm). Pfam domain content was used as a means to evaluate the performance of these algorithms, under the assumption that a poorly annotated genome will be globally depleted for domains relative to well-annotated genomes. (A) The average Pfam domain contents for six well annotated eukaryotic reference proteomes: *H. sapiens*, *M. musculus*, *D. melanogaster*, *C. elegans*, *A. thaliana*, and *S. cerevisiae*. These data provide an upper bound for the expected domain content of a newly sequenced genome. The region of the pie chart outlined in red indicates the percentage of genes containing a Pfam domain; these are further subdivided by GO molecular function. (B) The Pfam domain content of SNAP produced *ab initio *predictions compared to MAKER2-SNAP gene annotations for the *L. humile *genome. (C) The Pfam domain content of SNAP *ab initio *gene predictions and MAKER2-SNAP annotations in the *S. mediterranea *genome.

For reference purposes, Figure 1a provides high-level breakdown of domain contents for six reference genomes. On average, 68% of annotations in these six genomes contain a Pfam domain. For individual proteomes, the percent enrichment ranges from a low of 57% for *C. elegans *to a high of 78% for *M. musculus*.

To compare the performance of *ab initio *gene prediction algorithms to that of MAKER2 on second-generation genomes, we performed a proof-of-principle genome annotation of *Linepithema humile *(Argentine ant), and updated the genome-annotations of the *Schmidtea mediterranea *(flatworm) genome. For these analyses, we used the *ab initio *gene-predictor SNAP because it can be easily trained for new genomes using CEGMA (an HMM-based program that identifies and annotates a subset of highly conserved, universal eukaryotic genes). The gene models produced by CEGMA then serve as the initial training set for SNAP.

Even after training using the CEGMA gene models, we found that only 15% of SNAP *ab initio *gene predictions in *L. humile *contain a Pfam domain (Figure 1b). The MAKER2-generated proteome, by comparison, is highly enriched for domains (Figure 1b). In total, 56% of the *L. humile *MAKER2-supervised SNAP predictions contain Pfam domains.

We also performed a proof-of-principle annotation update of the *S. mediterranea *(flatworm) genome using transcriptome (mRNA-seq) data deposited in the NCBI Sequence Read Archive (SRP006000). MAKER2 uses its *GFF3 *pass-through capability to integrate mRNA-seq data into the annotation process. The mRNA-seq reads are first pre-processed by the user's algorithm of choice (i.e. TopHat, Cufflinks, etc.), and then converted to GFF3 files for use with MAKER2. MAKER2 provides easy to use utilities for converting the outputs of TopHat and Cufflinks to GFF3 files. The mRNA-seq data can then be used by MAKER2 in combination with *ab initio *gene predictions, EST and protein alignments to inform MAKER2's gene annotations. *For S. mediterranea*, when annotated using a version of SNAP that was trained using the CEGMA *S. mediterranea *gene models, only 6% of the SNAP gene predictions encode a Pfam domain (Figure 1c). By comparison, using the same version of SNAP, in conjunction with mRNA-seq data, 47% of the MAKER2 supervised SNAP gene predictions encoded a domain, demonstrating the ability of MAKER2 to use mRNA-seq data to improve the quality of the predictions.

Transcriptome data alone, however, is unlikely to capture all protein coding genes, and the 13,934 genes models produced by MAKER2 using only mRNA-seq data likely represent around 80% of all genes within this genome. When using all available ESTs together with mRNA-seq reads and the Uniprot/Swiss-Prot protein database (after excluding any existing *S. mediterranea *proteins), MAKER2 produced 17,883 gene models, 52% of which encode Pfam domains and have overlapping support from multiple data sources, indicating that that these 17,883 gene models represent a more complete model of the genome than can be obtained from transcriptome data alone.

Interestingly, not only are domain enrichments low for the *L. humile *and *S*. *mediterranea *SNAP *ab initio *predictions, the gene counts are also greatly inflated. Approximately 15,000 genes are expected for *S. mediterranea *and approximately 17,000 are expected for *L. humile *[6,9], both values well below the 63,622 and 420,224 gene predictions produced (respectively) when running SNAP on its own (outside of MAKER2). *Ab initio *gene-predictors have a recognized tendency to over predict[53], and as these results demonstrate, this tendency can be greatly exacerbated by the limited training data usually available for second generation genomes. In contrast, MAKER2's supervised SNAP-based gene counts are dramatically more consistent with the published expected counts. MAKER2 produced 13,785 gene annotations for *L. humile *and 17,883 for *S. mediterranea *(Note this is without further optimization and training of the gene-predictor SNAP).

These results stand in stark contrast to the great accuracy obtained by SNAP on model organism genomes presented in Table 1. They also make it clear that when training data are limited or of low quality, *ab initio *gene-predictors produce much more reliable results when supervised by MAKER2. This conclusion is also consistent with our earlier analyses where we annotated three model organism genomes using unmatched species parameter files (Table 2). Additionally MAKER2's use of mRNA-seq reads for annotating *S. mediterranea *demonstrates that these next-generation data can be effectively utilized by MAKER2 to greatly improve the final gene models.

### Annotation Edit Distance as a quality control metric

As the number of published genomes continues to expand, manual curation and validation of every annotation in every genome is simply infeasible. A more practical approach is to dedicate limited resources and manpower to curation and validation of only those gene annotations most in need of improvement. As we demonstrate below, MAKER2 provides an effective means for automated quality control of genome annotations. Even in cases where the administrators of genome databases have no plans to undertake manual curation, quality control measures are still desirable, as they provide a means for downstream users to judge the quality of an annotation before proceeding with experiments that depend upon the annotation's accuracy for success.

Identifying low quality gene annotations is a challenge not well addressed by existing annotation tools. While quality metrics such as sensitivity, specificity, and accuracy are convenient for evaluating the performance of gene-predictors, they presuppose the existence of reference gene models, which are not available for many newly sequenced genomes. Researchers working with second-generation annotations are thus in need of new quality control measures and annotation management tools.

To address this issue, we have adapted the Annotation Edit Distance (AED) measurement, developed by the Sequence Ontology, for use in MAKER2 as an annotation quality-control metric. AED is similar to the sensitivity and specificity measures used to judge gene-finder performance[55], but it differs in that no reference gene-model is used. Instead AED measures the distance between two annotations (each from a different releases of the same genome), and it makes no assumptions as to which one is the more correct. As originally formulated, AED provides a means to measures changes to a gene annotation from release to release. We have adapted AED for use in MAKER2 as a means to quantify the congruency between a gene annotation and its supporting evidence - EST, protein, and mRNA-seq alignments (see Implementation section for details). As we show in the analyses presented below, MAKER2's AED values provide a very useful measure for annotation quality control.

AED values are bounded between 0 and 1, with a value of 0 indicating an exact match between the intron exon coordinates of an annotation and its aligned evidence and 1 indicating no evidence support. Thus, database managers can use AED to sort gene models from best supported to worst in order to prioritize them for downstream manual review; MAKER2's AED values can also provide a rational basis for how much faith a researcher should put in an annotation before proceeding with downstream bench experiments where success will hinge upon the gene model being correct.

**Figure 2:**
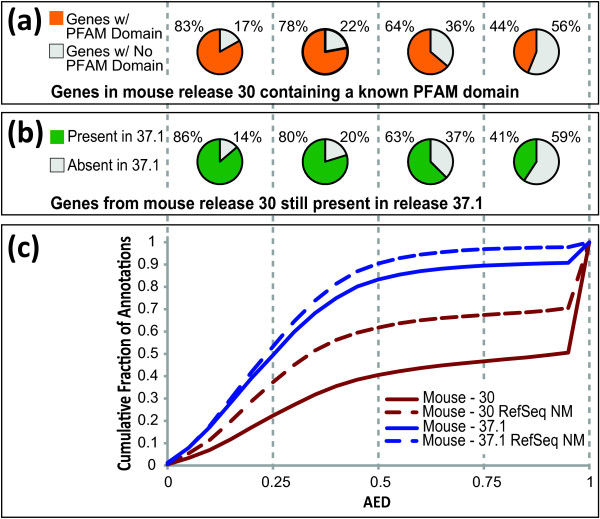
**Evaluating AED as a metric for annotation quality control**. Annotation Edit Distance (AED) provides a measurement for how well an annotation agrees with overlapping aligned ESTs, mRNA-seq and protein homology data. AED values range from 0 and 1, with 0 denoting perfect agreement of the annotation to aligned evidence, and 1 denoting no evidence support for the annotation. We evaluated the use of AED as a quality control metric by comparing MAKER2 produced AED scores for release 30 (2003) of the *M. musculus *genome to the AEDs for release 37.1 (2007). These data show how AED can be used to quantify improvements to the annotations between each release. (A) The Pfam domain content of *M. musculus *release 30 for genes found in each quartile of the MAKER2 AED distribution. Note that genes with low AEDs are highly enriched for domains. (B) The fraction of *M. musculus *genes from release 30 maintained/removed from subsequent release 37.1 for each MAKER2 AED distribution quartile. These data show how AED mirrors the independent curation decisions made by the mouse research community between 2003 and 2007. (C) The cumulative AED distributions of *M. musculus *release 30 and 37.1 demonstrate how AED quantifies improvements made between releases. The subset of genes with NM prefixes assigned by RefSeq (which indicates the highest level of annotation quality) is plotted separately to show that these independently identified 'gold-standard' gene annotations tend to have lower AED values in comparison to the genome as a whole.

As proof-of-principle, we compared MAKER2 produced AED scores for every annotation in release 30 of the *M. musculus *reference annotations (2003) to those of release 37.1 (2007) (Figure 2). We also performed the same analysis using reference annotations from human release 33 (2003) compared to human release 37.2 (2010) (Additional file 1 Figure S1). In order to perform these analyses, we first used MAKER2 to align EST and protein homology evidence against reference genome assemblies, and then compared these data to the mouse 30 and 37.1 and human 33 and 37.2 gene-models (thus producing AED scores for all annotations in the two datasets). We then plotted the cumulative distribution of AED for each dataset (Figure 2c and Additional file 1 Figure S1c).

As can be seen in mouse release 30 (Figure 2), there exists an abundance of genes in this early release with limited evidence support; in other words, a large portion of genes have high AED values). In contrast, for the more recent mouse release 37.1, the AED distribution is shifted toward lower AED (better) values. These two curves thus provide a high-level quantitative overview of the genome-wide improvements to the mouse gene-annotations between 2003 and 2007.

Notably, many of the release 33 mouse annotations nearly or completely lack support from EST and protein homology (as indicated by a spike of genes distributed around the AED value of 1). In contrast, for the more recent mouse release 37.1, there is nearly a complete elimination of the spike due to genes with AED scores near 1. This suggests that the earlier releases contained an abundance of false positive gene predictions that were deleted by release 37.1.

To further explore the extent to which AED scores are indicative of annotation quality, we also investigated the AED distribution of the highest quality subset of reference GenBank annotations from each of the mouse and human genome releases (the highest quality genes are those with NM prefixes assigned by RefSeq[57]). The RefSeq NM prefix provides us with an independently identified 'gold-standard' dataset of best quality annotations for comparison. For all releases, we see that the 'gold-standard' NM annotation datasets produce cumulative AED distributions that are shifted toward lower AED scores than the reference sets they are derived from (dotted lines in Figure 2c and Additional file 1 Figure S1c). This indicates that MAKER2 is able to verify the higher quality of these genes, and quantify the differences in quality, providing further support for the use of AED and MAKER2 as tools for annotation quality control.

We also investigated how well AED scores agreed with Pfam domain content. As can be seen in Figure 2a and Additional file 1 Figure S1a, AED scores accord well with domain content. In mouse release 30, for example, 87% of genes with AED scores from 0 to 0.25 contain a known domain, whereas only 44% of genes with an AED score ranging from 0.75 to 1.0 contain a domain. The trend is even more striking in human release 33 where only 15% of annotations with AED scores between 0.75 and 1.0 contain a domain, again suggesting there is a greater fraction of false positive gene predictions in that subset of genes (Additional file 1 Figure S1a). Tracking these annotations across releases supports this hypothesis: 86% of genes from human release 33 with AED scores between 0.75 and 1.0 are absent by release 37.2 (Additional file 1 Figure S1b). The same trend is observed in mouse: 59% of annotations in release 30 with AED scores between 0.75 and 1.0 were deleted by release 37.1 (Figure 2b). In comparison, only 14% of genes with AED scores between 0 and 0.25 were deleted between mouse release 30 and release 37.1.

Collectively, these results show that gene annotations judged to be of low quality by MAKER2 were also judged to be of low quality by GenBank and preferentially deleted (demonstrating that AED scores mirror the independent curation decisions made by the mouse and human research communities). These facts demonstrate the utility of MAKER2 as an annotation management tool.

### Re-annotation of existing genomes and legacy annotations

While there are a large number of second-generation genome projects underway, falling sequencing costs are also leading many researchers to revisit published genomes to improve gene models in light of new evidence, (such as mRNA-seq) or to take advantage of newer, more complete genome assemblies. There are also instances where researchers are sequencing individual strains/mutants of organisms where a published reference genome is already available or where multiple sets of legacy annotations exist and they wish to carry over annotations from the reference genome and merge them into a non-redundant consensus dataset. MAKER2 provides a simple method to perform these tasks via its external annotation pass-through mechanism that accepts as input any pre-existing genome annotations as well as aligned experimental evidence provided in a GFF3 formatted file.

When using this GFF3 pass-through mechanism, MAKER2 takes the user-provided gene models (from GFF3 files), aligns any additional experimental evidence against the genome (from standard FASTA files), and then calculates quality control statistics such as AED. If the user supplied MAKER2 with more than one legacy annotation dataset (i.e. multiple GFF3 files of alternate legacy annotations), MAKER2 chooses the one model most consistent with the evidence for each locus and carries it forward to produce a consensus (non-redundant) dataset. Researchers can also select to run *ab initio *gene-predictors (as is done for *de novo *annotation) in addition to providing a GFF3 file of legacy annotations. In this case, MAKER2 can produce new gene models for regions where the evidence suggests the existence of a gene that was not found in the legacy set, and with the help of the gene-finders MAKER2 will automatically update/revise the legacy annotations to better account for features suggested by aligned evidence.

**Figure 3:**
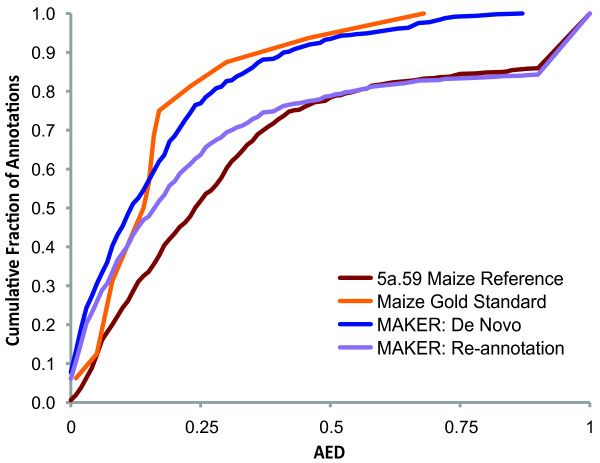
**Re-annotation of a portion of the Maize genome using MAKER2**. Annotation Edit Distance (AED) provides a measurement for how well an annotation agrees with its associated evidence (see text and Figure 1 for additional details). Shown are cumulative AED distributions for several Maize annotation datasets. Gold curve: AED distribution of high-quality 'gold standard' annotations in the benchmark region that are members of the J. Schnable and M. Freeling Classical Maize Genes List; These genes generally have the lowest AEDs. Red curve: all Maize gene models from the http://www.MaizeSequence.org 5a.59 Working Gene Set in the benchmark region; Blue curve: MAKER2's first pass, *de novo *annotations for the benchmark region; note that these genes generally have lower AEDs than the 5a.59 Working Gene Set (red curve). Purple curve: automatic MAKER2-based update/revision of the Maize 4a.53 Working Gene Set annotations. Note that the revised dataset now exceeds the quality of the 5a.59 Working Gene Set as judged by AED.

As proof-of-principle of MAKER2's model pass-through and re-annotation capabilities, we used the pipeline to process a 22 megabase region of maize inbred line B73 chromosome 4 together with version 5a.59 of the http://MaizeSequence.org Working Gene Set. For maize chromosome 4, we produced a *de novo *annotation gene set, a pass-through dataset (in which all reference annotations were maintained but tagged with evidence associations and AED values), and a re-annotation dataset (wherein MAKER2 was allowed to maintain or update reference annotations based on aligned experimental evidence). The cumulative distribution of AED scores for these three datasets was then graphed and is shown in Figure 3c. We also plotted the AED distribution of the high quality subset of reference annotations from the Maize Classical Gene List for comparison as an independently identified 'gold-standard' control dataset (Figure 3c, gold curve).

During re-annotation, 304 out of 493 version 5a.59 reference gene models were altered/updated to reflect features suggested by evidence alignments; 88 new gene models were produced for regions where the evidence suggested the existence of a gene but no model existed; and 189 reference gene models were left unchanged. A total of 89 of the unmodified reference gene models had no evidence support and were prioritized by MAKER2 for manual review as possible false positive annotations. Alterations to gene models during the re-annotation process caused the AED distribution curve for the re-annotation dataset (Figure 3c, purple curve) to shift towards lower AED values (better) relative to the reference annotation set (Figure 3c, red curve). This shift suggests that re-annotation using MAKER2 successfully brought gene models more in line with experimental evidence, thus improving their quality. A further comparison of both the re-annotation dataset and the unmodified reference dataset to the 'gold-standard' annotation set (Figure 3c, gold curve) supports this conclusion, as these high quality gene models also tend to be distributed around lower AED values (with more than 80% of 'gold-standard' annotations having AED values of < 0.2 compared to just 40% for the version 5a.59 reference annotation set). The spike in the AED distribution for both the unmodified reference dataset and the re-annotation dataset represents gene models that have little-to-no evidence support and are prioritized by MAKER2 for manual review. In comparison, the *de novo *annotation set (Figure 3c, blue curve) has an AED distribution shifted toward lower values than either the re-annotation or reference dataset; this is primarily due to the exclusion of unsupported gene models as the average AED for both the *de novo *and re-annotation datasets is identical when unsupported models are excluded (average AED of 0.17 in both).

### Managing existing annotation databases

With the proliferation of existing sequencing data, researchers have access to published genomes of multiple related species that may have been annotated using very different methods and to varying degrees of quality. Here, we evaluate how MAKER2's annotation pass-through option can be used to map cross-species data to multiple related genomes. We also explore how these data can be used to fuel downstream analyses such as cross-species orthology.

**Figure 4:**
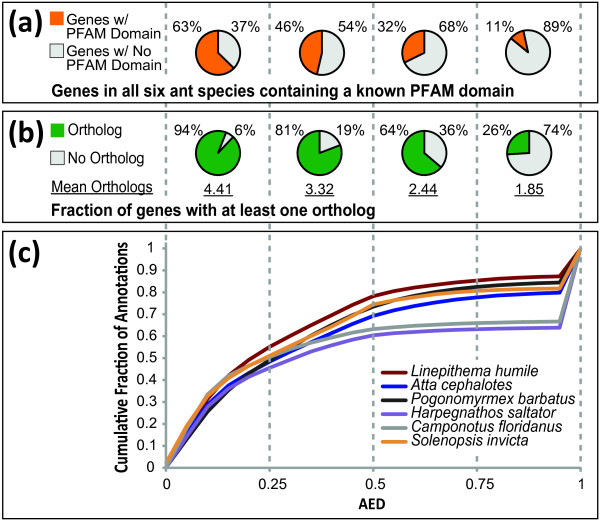
**MAKER2 as a management tool for existing genome annotations**. MAKER2 was used to add cross species homology evidence and AED values to six published ant species. These data show how MAKER2 can be used both to add new data to existing datasets and for downstream prioritization of genes in those datasets for further analysis and curation. (A) The Pfam domain content in each AED quartile. Genes receiving higher AED scores are less likely to contain a domain, thus prioritizing them as possible false positive gene predictions. (B) The percent of genes in each AED quartile having an orthologous protein in a related ant species with the average number of orthologs per gene (for the subset of orthologous genes) listed at the bottom. AED score is highly correlated with orthology. (C) The cumulative AED distribution for all six ant species. The spike of genes with AED score at or near 1 suggests potential false positive genes predictions rather than species-specific genes, as these annotations also generally lack EST support and Pfam domains; these gene models are first in MAKER2's list for manual review.

We used MAKER2 to map experimental evidence as well as reference annotations to six published ant genomes: *A. cephalotes*, *P. barbatus*, *L. humile*, *H. saltator*, *C. floridanus*, and *S. invicta*. The protein datasets provided to MAKER2 consisted of all proteins from UniProt/Swiss-Prot, *D. melanogaster*, *N. vitripennis *(wasp), *A. mellifera *(honey bee), and each of the previously mentioned published ant species (the individual species whose genome was being evaluated was always excluded from the protein dataset). We also included all Apocrita and Formicidae ESTs in dbEST with the EST dataset. Resulting cumulative AED distributions were then plotted for each ant species; average percent orthology and domain content were also evaluated for each quartile of the AED distribution (Figure 4).

Low AED scores indicate gene models with better agreement with evidence alignments, while higher values mean less evidence support. The cumulative distribution of AED scores for the six ant species can be seen in Figure 4c. For each ant species, there is a spike in the distribution curves around AED score 1. This spike represents genes that MAKER2 has prioritized for manual review. We see in Figure 4a that Pfam domain content is well correlated with AED score, and an average of 63% of genes with scores between 0 and 0.25 contain a Pfam domain compared to only 11% of genes with scores between 0.75 and 1.0. The low domain enrichment suggests that genes prioritized by MAKER2 are most likely false positive gene predictions--a conclusion supported by our earlier analyses of the mouse and human annotation datasets shown in Figure 2-- but there is also the potential that these represent novel genes with domains that would not be found in the Pfam domain database.

If we further expand our analysis to look at orthology among the ant species, we see that percent orthology between the six ant species is also well correlated to AED. Using reciprocal best blast hits as a rough definition of orthology, 94% of genes with AED scores between 0 and 0.25 have orthology to at least one protein in another ant species (on average there are 4.41 orthologous genes in other ant species that associate back to each of these), whereas only 26% of genes with AED scores between 0.75 and 1.0 have at least 1 ortholog in another ant species (for the genes here that have an ortholog there are only 1.85 orthologs that map back to them on average). Together with the domain analysis, the association of AED and orthology suggests that genes with AED scores near 1 are either recently evolved genes or false positive gene predictions; in either case, these genes should be targeted from manual review. Thus supporting the use of the AED statistic for quality control.

The ability of MAKER2 to align cross-species data to multiple genomes in this way demonstrates how MAKER2 can be used to generate common resources even when genomes are annotated using very different methods. Because all annotations and experimental evidence have been processed into a common format, they can now be easily loaded into downstream GMOD tools for analysis and data distribution. MAKER2 thus provides an efficient automated mechanism for research communities and organizations to manage shared genome database resources.

### High-throughput parallelization

**Figure 5:**
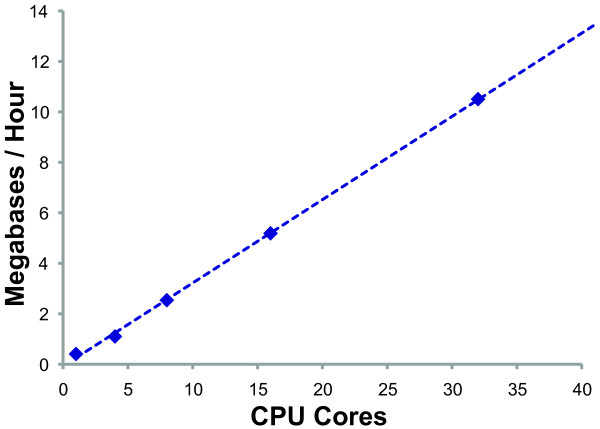
**MAKER2 scales to even the largest genomes**. MAKER2 was used to annotate a 10 megabase section of the *C. elegans *genome (NGASP dataset). The algorithm was parallelized using MPI on an increasing number of CPU cores. The results demonstrate how MAKER2 scales almost linearly with CPU number (with a slope of near 1). If we project our results forward to the entire *C. elegans *genome (~100 megabases), MAKER2 should take under 10 hours on 32 CPUs to complete; similarly, the human genome (~3 gigabases) would require fewer than 24 hours on 400 CPUs.

MAKER2 has been optimized to support high-throughput parallelization using Message Passing Interface (MPI), a distributed cluster communication protocol. To explore how data throughput in MAKER2 scales with processor usage, we annotated the 10 megabase NGASP dataset for *C. elegans *using an increasing number of processor cores (Figure 5). As can bee seen, data throughput scales linearly with processor usage: Annotating the entire 10 megabase dataset in just under 1 hour on 32 CPU cores; this means MAKER2 should be able to annotate the entire *C. elegans *genome in less than 10 hours using similar settings. Researchers with access to distributed computer clusters (300-3000 CPU cores) could expect to annotate even human-sized genomes (~2-3 gigabases) in less than 24 hours, while smaller fungal sized genomes (~40-80 megabases) could easily be annotated on laptop or desktop machines in the same time period. The scalability of data throughput for MAKER2 therefore allows researchers to process datasets of virtually any size or to process multiple datasets in a timely manner. MAKER2's high-throughput parallelization also provides a potential solution to the problem of annotating ultra large genomes such as pine trees, which have genomes in the 20-30 gigabase range[13].

It is important to note that much of MAKER2's computation time is spent aligning experimental evidence to the genome and analyzing the results. For this reason, the overall time required for genome annotation is expected to vary not only with genome length but also with the size of the input experimental evidence dataset. This upfront investment in computation time, however, provides enormous benefits downstream as all supplied EST reads, protein homology data, and gene predictions are available as searchable features in the final output. By loading MAKER2's output into GMOD tools like Chado[58], Galaxy[59], and GBrowse[60], researchers can quickly perform downstream analyses such as exploring protein orthology and analyzing sequence conservation. They can also identify cross-species changes in intron exon structures with the advantage of having all the information available directly from MAKER2's output without having to perform any additional computation.

## Conclusions

The performance of *ab initio *gene-predictors is heavily dependent on the availability of extensive training data. First-generation genome projects such as *D. melanogaster *thus benefitted greatly from the extensive knowledge of genes and gene structure that was already available before the genome projects even began. Unfortunately, second-generation (emerging model organisms) genomes generally lack pre-existing 'gold standard' gene models with which to train gene finders. These same projects, however, often do have on hand mRNA-seq data, a resource of obvious utility for annotation. Our results show that MAKER2 provides an easy means to integrate these and other data into the gene prediction and annotation process, resulting in dramatic improvements to annotation quality even when gene-finders are poorly trained.

By aligning evidence from ESTs, mRNA-seq, and protein homology, MAKER2 also provides a convenient way to add these types of experimental data to new and existing annotation datasets for purposes of quality control, and as a means to update and revise legacy annotation datasets automatically. As proof-of-principle, we demonstrated that MAKER2 was able to prioritize genes for review from mouse release 30 and human release 33. This prioritization is well correlated with the deletion and revision of the same genes in subsequent mouse release 37.1 and human release 37.2, indicating that MAKER2's AED-based prioritization method closely emulates the quality control decisions used for these genomes. Likewise, our re-annotation of portions of the Maize genome demonstrates the ability of MAKER2 to automatically revise and update existing genome annotations. MAKER2 thus provides an automated means of quality control for both new and existing genome annotations; this in turn will allow researchers to make more informed decisions when designing experiments whose success is dependent upon the correctness of the annotation as incorrect annotations poison every experiment that uses them.

## Availability and requirements

MAKER2 is a Perl-based application, is freely available for academic use. Source code, documentation and a user tutorial are available at

http://www.yandell-lab.org/software/maker.html

Links to a bug tracker and users' email list are also available on the download page.

## Authors' contributions

CH and MY conceived the study and wrote the manuscript. CH carried out experiments and wrote software for the analyses. All authors read and approved the final manuscript.

## Supplementary Material

Additional file 1 **Supplementary tables and figures**. Contains Supplementary Tables 1, 2, and 3 as well as Supplementary Figure 1. Click here for file
