# Supplementary material for: MAKER2: an annotation pipeline and genome-database management tool for second-generation genome projects
Source: BMC Bioinformatics. 2011 Dec 22;12:491. doi: 10.1186/1471-2105-12-491 (PMC3280279; doi:10.1186/1471-2105-12-491)
Supplement: Additional file 1 — Supplementary tables and figures. Contains Supplementary Tables 1, 2, and 3 as well as Supplementary Figure 1. [file 1471-2105-12-491-S1.PDF]

Supplementary Table 1. Gene model sensitivity/specificity for *ab initio* prediction and genome annotation programs

	Ab Initio Predictions			MAKER2 Annotations		
Arabidopsis thaliana	Augustus	GeneMark	SNAP	Augustus	GeneMark	SNAP
Gene Sensitivity	35.91%	29.41%	15.67%	23.72%	28.43%	26.33%
Gene Specificity	30.23%	19.05%	13.53%	36.50%	35.07%	34.17%
Transcript Sensitivity	29.85%	24.06%	12.82%	19.61%	23.33%	21.61%
Transcript Specificity	30.23%	19.05%	13.53%	33.41%	35.07%	34.09%
Exon Sensitivity	70.79%	68.06%	58.86%	63.30%	63.39%	62.69%
Exon Specificity	63.27%	54.56%	53.93%	72.31%	75.80%	74.87%
Nucleotide Sensitivity	85.49%	83.08%	76.38%	73.01%	70.91%	71.93%
Nucleotide Specificity	68.58%	66.27%	63.17%	88.05%	87.87%	88.60%
Drosophila melanogaster						
Gene Sensitivity	19.28%	7.62%	8.30%	17.10%	18.78%	17.60%
Gene Specificity	22.41%	7.61%	9.06%	23.34%	21.72%	21.72%
Transcript Sensitivity	14.07%	5.44%	6.08%	12.42%	13.60%	12.78%
Transcript Specificity	22.41%	7.61%	9.06%	21.23%	21.72%	21.69%
Exon Sensitivity	57.37%	50.18%	49.17%	53.05%	51.88%	52.77%
Exon Specificity	65.36%	44.44%	44.85%	64.07%	64.17%	64.21%
Nucleotide Sensitivity	76.67%	69.07%	70.18%	69.77%	66.33%	66.92%
Nucleotide Specificity	75.49%	64.00%	68.40%	83.07%	80.98%	81.74%
Caenorhabditis elegans						
Gene Sensitivity	36.48%	29.95%	22.89%	24.57%	24.70%	25.39%
Gene Specificity	41.76%	25.04%	18.01%	34.88%	24.03%	29.87%
Transcript Sensitivity	32.59%	25.68%	20.21%	21.79%	21.76%	22.30%
Transcript Specificity	41.76%	25.04%	18.01%	33.47%	24.03%	29.76%
Exon Sensitivity	71.43%	72.79%	65.41%	62.01%	61.35%	63.48%
Exon Specificity	77.81%	64.96%	57.34%	75.18%	68.70%	68.90%
Nucleotide Sensitivity	87.34%	92.50%	87.31%	80.13%	80.64%	84.64%
Nucleotide Specificity	89.23%	83.67%	82.89%	94.14%	91.94%	92.32%

Supplementary Table 2. Gene model sensitivity/specificity when using unmatched species parameter files

	Ab Initio Predictions			MAKER2 Annotations		
Arabidopsis thaliana	Augustus	GeneMark	SNAP	Augustus	GeneMark	SNAP
Gene Sensitivity	3.45%	4.54%	2.72%	9.36%	3.92%	14.34%
Gene Specificity	7.40%	4.27%	3.93%	17.61%	7.90%	22.34%
Transcript Sensitivity	2.94%	3.78%	2.18%	7.82%	3.38%	11.92%
Transcript Specificity	7.40%	4.27%	3.93%	17.61%	7.90%	22.34%
Exon Sensitivity	19.51%	10.82%	9.48%	38.04%	13.54%	46.86%
Exon Specificity	41.91%	22.19%	27.67%	68.57%	44.19%	73.35%
Nucleotide Sensitivity	42.09%	30.99%	19.79%	45.89%	25.45%	54.82%
Nucleotide Specificity	73.60%	66.24%	67.88%	91.22%	90.46%	92.71%
Drosophila melanogaster						
Gene Sensitivity	18.17%	18.17%	17.60%	13.94%	13.14%	25.03%
Gene Specificity	4.96%	3.94%	3.43%	14.36%	11.98%	23.30%
Transcript Sensitivity	13.37%	13.14%	12.35%	10.29%	9.73%	17.99%
Transcript Specificity	4.96%	3.94%	3.43%	14.36%	11.98%	23.30%
Exon Sensitivity	40.12%	36.00%	24.24%	39.18%	33.34%	44.28%
Exon Specificity	21.12%	16.50%	15.63%	47.02%	46.13%	63.09%
Nucleotide Sensitivity	82.45%	81.32%	53.76%	66.82%	62.39%	61.44%
Nucleotide Specificity	52.49%	51.69%	44.07%	80.74%	83.27%	87.44%
Caenorhabditis elegans						
Gene Sensitivity	4.39%	8.13%	12.09%	4.80%	8.29%	24.84%
Gene Specificity	5.27%	6.99%	9.29%	7.57%	11.29%	29.94%
Transcript Sensitivity	4.18%	7.97%	11.64%	4.32%	8.10%	22.22%
Transcript Specificity	5.28%	7.00%	9.29%	7.05%	11.29%	29.59%
Exon Sensitivity	20.19%	23.63%	27.98%	28.36%	26.94%	55.44%
Exon Specificity	36.46%	36.38%	42.90%	48.68%	51.89%	70.84%
Nucleotide Sensitivity	52.64%	55.84%	56.64%	56.46%	52.49%	75.92%
Nucleotide Specificity	79.72%	78.67%	79.84%	92.17%	91.35%	94.12%

Supplementary Table 3. PFAM domain content of six reference genomes and division by GO molecular function

	Homo sapiens	*Mus musculus*	*Drosophila melanogaster*	*Caenorhabditis elegans*	*Arabidopsis thaliana*	*Saccharomyces cerevisiae*	Mean	Variance	Standard Deviation	Combined Species	Normalize for domain overlap and non-domain containing gene counts
Gene Count	20584	22758	13903	22240	27416	5861	-	-	-	112762	-
Genes w/ PFAM Domain	15443	17734	9243	12782	17945	4051	-	-	-	77198	-
Genes w/ GO Molecular Function	9962	11033	6069	7547	11634	2431	-	-	-	48676	-
% Genes w/ PFAM Domain	75.02%	77.92%	66.48%	57.47%	65.45%	69.12%	68.58%	0.00533	7.30%	**68.46%**	-
GO Molecular Function (% of PFAM subset):											
molecular function	0.01%	0.01%	0.02%	0.00%	0.00%	0.00%	0.01%	8.25E-09	0.01%	0.01%	**0.00%**
protein transporter activity	0.26%	0.25%	0.36%	0.13%	0.30%	0.44%	0.29%	1.16E-06	0.11%	0.27%	**0.14%**
translation regulator activity	0.21%	0.24%	0.32%	0.23%	0.34%	0.52%	0.31%	1.32E-06	0.11%	0.28%	**0.15%**
antioxidant activity	0.17%	0.17%	0.24%	0.31%	0.64%	0.22%	0.29%	3.10E-06	0.18%	0.31%	**0.16%**
signal transducer activity	0.62%	0.58%	0.30%	0.39%	0.51%	0.22%	0.44%	2.52E-06	0.16%	0.49%	**0.25%**
isomerase activity	0.45%	0.37%	0.55%	0.43%	0.59%	0.94%	0.55%	4.21E-06	0.21%	0.50%	**0.26%**
motor activity	0.64%	0.66%	0.63%	0.33%	0.45%	0.25%	0.49%	3.15E-06	0.18%	0.52%	**0.27%**
lyase activity	0.43%	0.44%	0.75%	0.67%	0.71%	0.67%	0.61%	1.95E-06	0.14%	0.59%	**0.31%**
helicase activity	0.80%	0.77%	0.98%	0.84%	0.92%	2.15%	1.08%	2.82E-05	0.53%	0.92%	**0.48%**
electron carrier activity	0.67%	0.90%	1.29%	0.88%	2.26%	0.72%	1.12%	3.62E-05	0.60%	1.21%	**0.63%**
ligase activity	1.18%	1.14%	1.54%	1.06%	1.28%	2.10%	1.38%	1.51E-05	0.39%	1.27%	**0.66%**
channel activity	1.66%	1.45%	1.87%	2.04%	0.37%	0.22%	1.27%	6.08E-05	0.78%	1.33%	**0.69%**
transporter activity	1.39%	1.31%	2.13%	1.57%	2.27%	2.30%	1.83%	2.07E-05	0.45%	1.75%	**0.91%**
structural molecule activity	1.28%	1.62%	1.50%	2.05%	2.11%	3.97%	2.09%	9.56E-05	0.98%	1.85%	**0.97%**
enzyme regulator activity	2.71%	2.59%	2.36%	1.42%	1.44%	1.14%	1.94%	4.70E-05	0.69%	2.05%	**1.07%**
catalytic activity	1.66%	1.53%	2.15%	2.11%	2.51%	4.22%	2.36%	9.53E-05	0.98%	2.10%	**1.10%**
receptor activity	2.07%	3.08%	1.98%	3.83%	0.90%	0.02%	1.98%	1.92E-04	1.39%	2.20%	**1.15%**
ion transmembrane transporter activity	2.92%	2.63%	3.55%	3.60%	1.57%	1.97%	2.71%	6.76E-05	0.82%	2.68%	**1.40%**
kinase activity	3.90%	3.76%	3.15%	4.18%	6.58%	3.80%	4.23%	1.44E-04	1.20%	4.43%	**2.32%**
oxidoreductase activity	3.25%	3.46%	5.34%	4.00%	6.84%	5.26%	4.69%	1.89E-04	1.38%	4.62%	**2.41%**
transcription regulator activity	5.24%	4.73%	3.60%	4.80%	4.67%	3.88%	4.49%	3.82E-05	0.62%	4.64%	**2.43%**
transferase activity	3.98%	3.78%	4.68%	5.06%	5.47%	5.88%	4.81%	6.78E-05	0.82%	4.65%	**2.43%**
hydrolase activity	8.59%	8.38%	12.32%	9.27%	9.52%	9.53%	9.60%	2.01E-04	1.42%	9.38%	**4.91%**
nucleic acid binding	12.73%	11.51%	10.58%	9.40%	11.18%	11.87%	11.21%	1.31E-04	1.14%	11.23%	**5.87%**
protein binding	17.98%	16.41%	11.74%	11.56%	11.23%	8.64%	12.93%	1.24E-03	3.53%	13.72%	**7.17%**
binding	22.39%	20.55%	19.47%	18.51%	22.44%	22.39%	20.96%	2.93E-04	1.71%	20.99%	**10.98%**
GO function unknown	35.49%	37.79%	34.34%	40.96%	35.17%	39.99%	37.29%	7.48E-04	2.74%	36.95%	**19.32%**

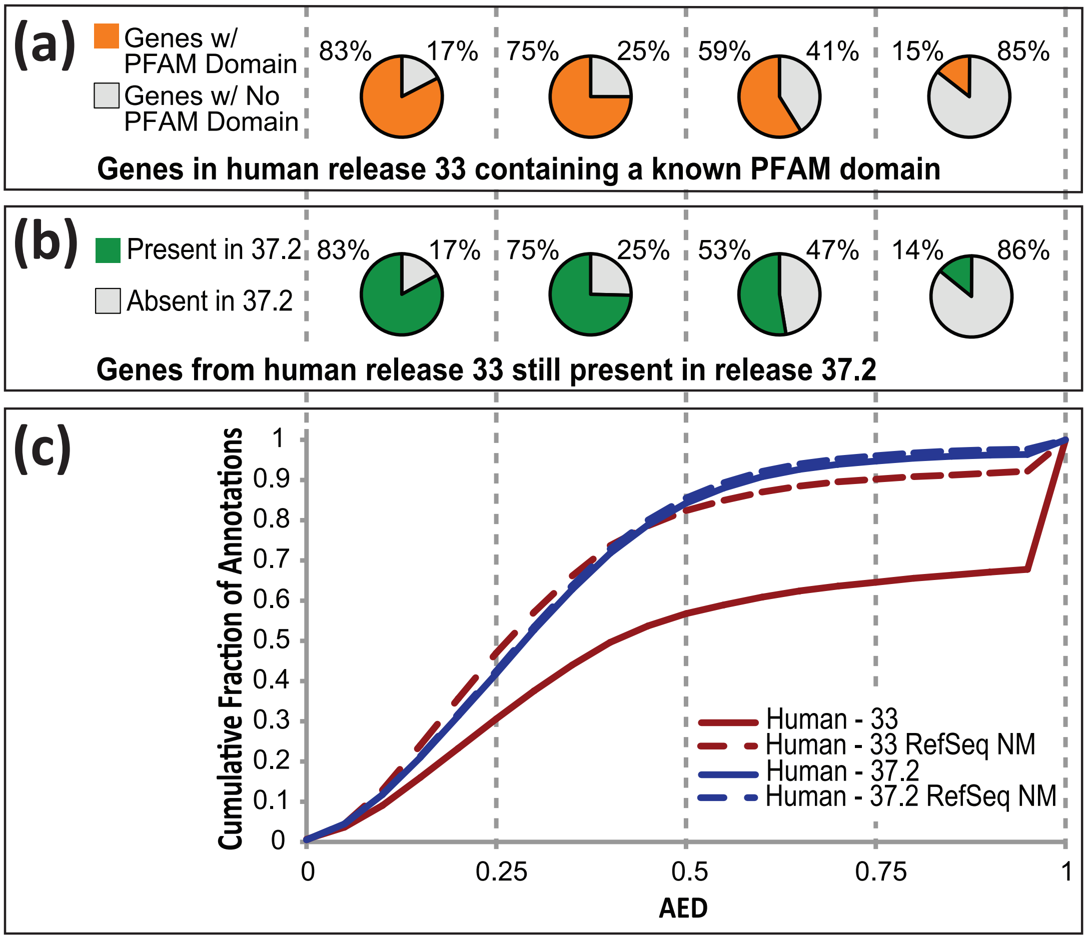

Supplementary Figure 1. AED evaluation of *Homo sapiens* reference annotations. Annotation Edit Distance (AED) provides a measurement for how well an annotation agrees with its associated evidence, as regards its overlap relative to aligned ESTs, mRNA-seq and protein homology data. AED values range from 0 to 1, with 0 denoting perfect agreement of the annotation to aligned evidence, and 1 denoting no evidence support for an annotation. We evaluated the use of AED as a quality control metric by comparing MAKER2 produced AED scores for release 33 (2003) of the *H. sapiens* genome to the AEDs for release 37.2 (2010). These data show how AED can be used to quantify improvements to annotations between releases. (A) The Pfam domain contents of *H. sapiens* release 33 for genes found in each quartile of the MAKER2 AED distribution. Note that genes with low AEDs are highly enriched for domains. (B) The fraction of *H. sapiens* genes from release 33 maintained/removed from subsequent release 37.2 for each MAKER2 AED distribution quartile. These data show how AED mirrors the independent curation decisions made by the human genetics research community between 2003 and 2010. (C) The cumulative AED distributions of *H. sapiens* release 33 and 37.2 demonstrate how AED quantifies improvements made between releases. The subset of genes with NM prefixes assigned by RefSeq (which indicates the highest level of annotation quality) is plotted separately to show that these independently identified 'gold-standard' gene annotations tend to have lower AED values in comparison to all genes as a whole.
